# Could Zika virus emerge in Mainland China? Virus isolation from nature in *Culex quinquefasciatus*, 2016

**DOI:** 10.1038/emi.2017.80

**Published:** 2017-11-01

**Authors:** Song Song, Yuanyuan Li, Shihong Fu, Hong Liu, Xiaolong Li, Xiaoyan Gao, Ziqian Xu, Guoping Liu, Dingming Wang, Zhenzao Tian, Jingzhu Zhou, Ying He, Wenwen Lei, Huanyu Wang, Bin Wang, Xiaoqing Lu, Guodong Liang

**Affiliations:** 1 Qingdao University, Qingdao 266071, China; 2 State Key Laboratory of Infectious Disease Prevention and Control, National Institute for Viral Disease Control and Prevention, Chinese Center for Disease Control and Prevention, Beijing 102206, China; 3 Collaborative Innovation Center for Diagnosis and Treatment of Infectious Diseases, Hangzhou 310058, China; 4 Shandong Provincial Research Center for Bioinformatic Engineering and Technique, School of Life Sciences, Shandong University of Technology, Zibo 255049, China; 5 Center for Disease Control and Prevention of Shenyang Command, Shenyang 110034, China; 6 Guizhou Province Center for Disease Control and Prevention, Guiyang 550004, China

**Dear Editor,**

Zika virus (ZIKV) was first isolated from a rhesus monkey in Uganda in 1947.^1^ Human infection with ZIKV often causes self-limiting symptoms with fever. A ZIKV outbreak in humans occurred in 2007 on Yap Island, in Micronesia, in the western Pacific,^2^ and large outbreaks have swept through Brazil, Columbia, and other countries in South America since 2015, resulting in hundreds of thousands of infectious cases. Infectious cases of ZIKV and imported cases have been reported in dozens of countries around the world.^3^ Moreover, many cases of microcephaly and Guillain-Barre syndrome associated with ZIKV infection have been identified. The World Health Organization (WHO) has declared these large-scale ZIKV infections to be a Public Health Emergency of International Concern, due to the huge public health burden they cause.^4^ Previous studies have suggested that *Aedes* mosquitoes are the major vectors of ZIKV. ZIKV in South America was spread by *Aedes aegypti*.^5^ In this study, a strain of ZIKV (GZDJ1685) was isolated from specimens of *Culex quinquefasciatus* collected in the southern China. This is not only the first report of ZIKV isolation in mainland China in nature, but also the first time that ZIKV has been isolated from this species in East Asia.

In mid-August 2016, a survey of mosquitoes and mosquito-borne viruses was carried out in Dejiang County, Guizhou Province, in southwestern China (108° 02′24″ E, 28° 15′19″ N latitude). Mosquito specimens were classified by morphology and stored in liquid nitrogen, with 50 mosquitoes per pool. Each pool of mosquito samples was ground and centrifuged at 13 000 r/min at 4 °C for 30 min. The supernatant was inoculated into tissue culture cells in parallel, and cytopathic effects (CPEs) were observed daily, using a microscope.^6^ Virus RNA was extracted from cultures that were positive for CPE to prepare viral cDNA. Then the cDNA was used as a template to detect the genes of *Alphavirus*, *Flavivirus* and *Bunyavirus*.^6, 7^ Sequences of positive products were assembled using SeqMan in the DNASTAR software package and then aligned with the relevant sequences downloaded from GenBank. A phylogenetic analysis of viral genes was conducted using Mega software version 6.0.^6^ A total of 5795 mosquitoes were collected, of which 2540 were *Anopheles sinensis*, 1700 were *Culex quinquefasciatus*, 1530 were *Armigeres subbalbeatus* and 25 were *Culex fuscanus*. After grinding samples, inoculating supernatants into cells, four virus isolates (GZDJ1608, GZDJ1609, GZDJ1648 and GZDJ168585) that were stable in C6/36 and BHK cells were obtained. They were all isolated from *Culex quinquefasciatus*. Molecular identification showed negative results with *Bunyavirus* and *Alphavirus*-specific primers, but positive products were obtained with *Flavivirus*-specific amplification primers. Sequencing and molecular analyses of PCR-positive products indicated that GZDJ1608, GZDJ1609 and GZDJ1648 were Japanese encephalitis virus (JEV). Phylogenetic analyses based on the JEV E gene showed that these three isolates were genotype 1 (data not shown).

GZDJ1685 had CPEs in BHK and C6/36 cells at 48 h. The nucleotide sequence of the amplified product of GZDJ1685 extracted using *Flavivirus*-specific primers was highly homologous with ZIKV. The sequence of the coding region of GZDJ1685 (GenBank No. MF099651) was obtained using an amplification primer of the whole ZIKV genome sequence.^7^ Nucleotide and amino acid homology was greatest between GZDJ1685 and H/PF-2013, isolated from ZIKV-infected patients in French Polynesia in 2013 (99.5% and 99.5%, respectively). Phylogenetic analyses (Figure 1) showed that GZDJ1685 was located in the Asian branch of ZIKV on the phylogenetic tree, along with ZIKV strains isolated from Brazil (2015), Puerto Rico (2015) and Yap Island (2007).

Dejiang County is located in the southwestern area of China’s Yunnan-Guizhou Plateau, and it has an average elevation of 1000–1500 m. It has a warm and humid climate; the annual average temperature ranges from 13 °C to 17 °C, and it has a frost-free period of 295 days with ample sunshine and rainfall. The collection points were distributed in mountainous areas where residents build houses and terraces on the hillside. Corn and sorghum are the main agricultural products. The local farmers keep livestock such as pigs, sheep, chickens and ducks near their dwellings. Dejiang County is traditionally an endemic region for JE,^8^ but no ZIKA case and *Aedes aegypti* have been reported in the local region.

Phylogenetic analyses of the viral genome sequence revealed that the ZIKV (GZDJ1685) isolated from *Culex quinquefasciatus* in China belongs to genotype 2 in the Asian evolutionary branch of ZIKV (Figure 1), as do ZIKVs isolated from Brazil, Haiti, Puerto Rico, French Polynesia and Thailand during the period of 2013 to 2016, suggesting that the strain isolated from *Culex quinquefasciatus* was derived from the Asian population of ZIKV.

Although ZIKV is an *Aedes* mosquito-transmitted virus, it has been detected or isolated from a variety of mosquito species in nature. There were 31 ZIKVs isolated from mosquitoes collected in Seychelles, 28 of which were isolated from 10 species of *Aedes* and the other three were isolated from *Ma. uniformis*, *Cx. perfuscus* and *An. coustani*, respectively.^9^ Early in the 1950s, a laboratory study has found that *Culex quinquefasciatus* was a potential vector for ZIKV.^1^ Recent studies further revealed that ZIKV can be detected in the salivary glands of *Culex quinquefasciatus* 7–15 days after its infection with ZIKV in the laboratory.^10, 11^ In addition, PCR-positive ZIKV has been identified in the brain tissues of mice bitten by ZIKV-infected mosquitoes of *Culex quinquefasciatus*.^11^ Furthermore, a total of 1496 *Culex quinquefasciatus* mosquitoes and 408 *Ae. aegypti* mosquitoes were collected from different sites in Brazil during the ZIKV epidemic period (February to May) in 2016. From 270 pools of *Culex quinquefasciatus* and 117 pools of *Ae. aegypti* assayed by RT-qPCR, three pools of *Culex quinquefasciatus* and two pools of *Ae. aegypti* were detected as PCR-positive for ZIKV. And two ZIKVs were isolated from *Culex quinquefasciatus* and were stable in Vero cells.^12^ Taken together, these results suggest that *Culex quinquefasciatus* has the ability to not only help replicate ZIKV but also spread ZIKV to other animals through bites.

During the outbreaks of ZIKV spread in Brazil and other countries in Americas since 2015, imported cases infected with ZIKV have also been reported in China, but there were no secondary cases discovered.^13^ Previous field and laboratory studies on mosquito-borne arbovirus in China did not detect and isolate ZIKV in China in last two decades either.^14^ Thus, the result of this study is the first report of ZIKV isolation in nature not only in mainland China, but also in East Asia. And it will bring new challenges for the prevention and control of ZIKV in China. For example, will ZIKV infection exist in local wild animals, especially in non-human primates at the place where this *Culex quinquefasciatus* carried ZIKV was isolated? Are there any ZIKVs carried by *Aedes* mosquitoes, especially the domestic form of *Ae. aegypti*, spread in that area? Recent studies have suggested the domestic form of *Ae. aegypti* played an important role in the emergence of *Aedes* transmitted arboviruses and associated arboviral diseases.^15^ What is the infection status of the local human population? To answer these questions and evaluate the burden of disease and public health impact, additional detection and monitoring of ZIKV and its infection in China are necessary to be carried out thoroughly, including the use of seroprevalence studies for evidence of exposure to ZIKV.

## Figures and Tables

**Figure 1:**
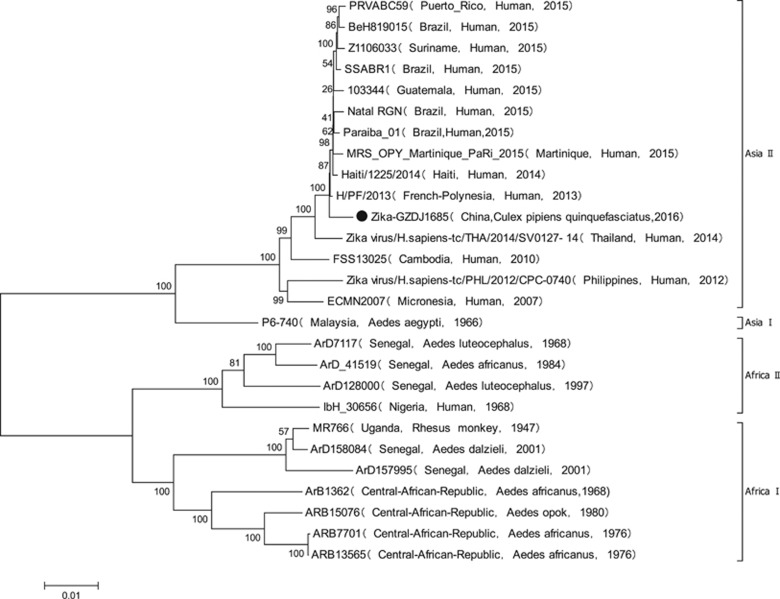
Phylogenetic analysis based on the coding region sequences of ZIKV (GZDJ1685) isolated from *Culex quinquefasciatus*.
